# Defining benchmarks for postoperative mobilization based on the recommendations of the Enhanced Recovery After Surgery (ERAS) program for liver surgery: a prospective study

**DOI:** 10.1007/s00464-025-12194-2

**Published:** 2025-09-17

**Authors:** Pia F. Koch, Simon Moosburner, Nathanael Raschzok, Robert Oehring, Philipp Brunnbauer, Alexandra Zühlke, Marlen Breitkreutz, Phillip Pfeffer, Karl H. Hillebrandt, Wenzel Schöning, Johann Pratschke, Igor M. Sauer, Jens Neudecker, Felix Krenzien

**Affiliations:** 1 Department of Surgery, Experimental Surgery, Charité – Universitätsmedizin Berlin, corporate member of Freie Universität Berlin and Humboldt-Universität zu Berlin, Campus Charité Mitte | Campus Virchow-Klinikum, Augustenburger Platz 1, 13353 Berlin, Germany; 2 BIH Charité Clinician Scientist Program, Berlin Institute of Health at Charité – Universitätsmedizin Berlin, BIH Biomedical Innovation Academy, Charitéplatz 1, 10117 Berlin, Germany

**Keywords:** Enhanced Recovery after Surgery (ERAS), Textbook outcome after liver surgery, Minimally invasive liver surgeries (MILS)

## Abstract

**Background:**

Early mobilization is a core component of the Enhanced Recovery After Surgery (ERAS) protocol, aiming to accelerate recovery and reduce postoperative complications. In the context of liver surgery, early mobilization is supposed to be associated with improved outcomes, yet the specific influence of timepoint and duration of mobilization remains unexplored. This study seeks to evaluate benchmarks of early mobilization within a structured ERAS program according to the ERAS guidelines to establish evidence-based recommendations for its timing and duration.

**Methods:**

A prospective observational study was conducted on 1,076 patients undergoing liver surgery within an ERAS protocol that strictly followed the official ERAS Society recommendations. Mobilization data were collected from postoperative day (POD) 0 through POD 3 for specific liver resections, such as hepatectomy, limited liver resections, and comparisons between open (OR) and minimally invasive liver surgery (MILS). Two patient groups were defined based on the presence or absence of a textbook outcome (TO): Patients who achieved a TO were defined as no complications, no prolonged hospital stay, no readmissions, and no mortality (*n* = 261) vs. Patients who did not (*n* = 715; control group).

**Results:**

Patients without complications, across all types of liver resections, were mobilized on POD 1, POD 2, and POD 3 for a median of 2 h (IQR 1–4), 4 h (2–6), and 5 h (4–7), respectively. This duration was significantly longer than in patients who experienced any type of postoperative complications (*p* < 0.001). A MILS right hepatectomy was associated with significantly shorter mobilization times on POD1 to POD3—2 h (1–3), 3 h (2–4), and 4 h (3–6), respectively—compared to a MILS segmentectomy, which showed mobilization times of 2 h (2–4), 4 h (3–6), and 6 h (4–7). In general, mobilization was 2 h longer in patients that underwent MILS in comparison to OR (*p* < 0.001). Shorter surgeries starting earlier in the day facilitated early mobilization on POD 0 (*p* < 0.001).

**Conclusion:**

Our findings highlight the importance of postoperative mobilization and define cut-offs for the type of liver resection from easy to complex. However, applying a uniform cutoff for all types of liver resections appears more than questionable, given the procedure-specific differences in postoperative mobilization.

Modern approaches in interdisciplinary postoperative patient care have proven effective in reducing complications and enhancing outcomes across various surgical specialties [1, 2]. The Enhanced Recovery After Surgery (ERAS program) is a multimodal, evidence-based, and patient-centered approach that revolutionizes perioperative care through an innovative, multidisciplinary international treatment pathway, emphasizing both optimal perioperative management and the importance of early postoperative mobilization. The ERAS society published consensus protocols for patients undergoing liver surgery and updated them in 2022 [3, 4]. Preoperative strategies within ERAS, such as optimizing nutrition and reducing alcohol and nicotine use, are combined with an educational and motivational talk by the ERAS nurses. In addition, early mobilization is supported and facilitated during the postoperative phases [5, 6].

Indeed, mobilization is recognized as a cornerstone of improving recovery and is a key component of ERAS protocol [7]. Mobilization is discussed in reducing postoperative complications, including those related to pulmonary and cardiac functions and surgical site infections [5], reducing overall length of hospital stay and a quicker return to normal activities, enhancing the patient’s overall quality of life during recovery [8–10]. However, there is little to no data on early mobilization after liver resection [11]. Consequently, the guidelines of the ERAS society state there is no evidence to give any recommendations regarding the optimal duration of mobilization [3]. Our goal is to address this gap in the guidelines by analyzing mobilization data following liver surgery and dissecting possible differences between specific liver resections like hepatectomy, limited liver resections, or open (OR) vs. minimally invasive liver surgery (MILS).

## Methods

This prospective observational study was approved by the local ethics committee under application numbers EA2/108/18 and EA4/153/18 and was registered with the German Clinical Trials Register (DRKS00030908). The study was performed following the guidelines of the Declaration of Helsinki. All study participants gave written informed consent to participate in the study and to the processing of personal data. From August 2018 to March 2024, 1,076 patients undergoing elective liver resection within an ERAS program at the Department of Surgery, Campus Virchow-Klinikum, Charité – Universitätsmedizin Berlin, were included.

### ERAS setup

The structure of the ERAS team at Charité is shaped by multiple stakeholders, including the ERAS nursing team, nursing scientists, and ward managers, as well as a lead ERAS physician from surgery, anesthesiologists, and physiotherapists. This team is responsible for implementing the 28 ERAS protocol items and consists of surgeons, anesthesiologists, and dedicated ERAS study nurses who oversee both the program and patient care [12]. The ERAS protocol strictly followed the official ERAS society recommendations and is documented with the ERAS Interactive Audit System (EIAS, Encare, Stockholm, Sweden [12]). A dedicated ERAS nurse was actively involved in the care process, coordinating activities and supporting patients both in the intensive care unit (ICU) and on the normal ward. In addition to standard preoperative patient education sessions in the ERAS consultation, an education group led by an ERAS nurse and a sports therapist was introduced on the day of admission. A visual mobilization pathway with pictograms at the ward level (similar to a fitness trail) to encourage patient movement and activity was established. Physiotherapy played a crucial role in mobilizing patients early postoperatively, fostering quicker recovery. Moreover, patient diaries were utilized to enhance patient engagement and track individual progress throughout their hospital stay. Monthly feedback on mobilization data to inpatient wards, provided through “One-Minute Wonder” sessions or audits, ensured continuous data feedback. The standard care included preadmission patient work-up, including blood analysis and liver function tests in case of major liver resections. Furthermore, preoperative sedative medication was allowed if desired by the patients, and intraoperatively, drains were placed as desired by the surgeon. No routine low-molecular weight heparin was administered preoperatively.

### Inclusion and exclusion criteria

Patients who were at least 18 years old and underwent elective liver resection within the ERAS protocol were included. Written informed consent was obtained from all patients prior to treatment. Patients were excluded from the analysis in cases of intraoperative decision against liver resection, such as cases with peritoneal carcinosis, or in cases of simultaneous resection of another organ during the same surgery (e.g., colorectal resection).

The applied ERAS protocol was based on the first iteration of the official ERAS guidelines for liver surgery of the ERAS Society from 2016 and was adapted according to the revised 2022 Guidelines [13, 14]. The ERAS protocol was implemented at our department in 2019 and supervised by an ERAS core team, consisting of surgeons, anesthesiologists, physiotherapists, nursing staff, and ERAS nurses [12]. Based on the ERAS measures of the EIAS, standard operating procedures as well as patient information brochures and patient diaries were created. The implementation of the ERAS protocol comprised specific interdisciplinary ERAS training of the staff and regular audit meetings.

### Patients

A Textbook Outcome (TO) following liver surgery was defined based on the following criteria: no postoperative complications, no prolonged hospital stay (defined as a postoperative length of stay [LOS] exceeding the 75th percentile of the cohort, consistent with previous ERAS literature), no readmissions, and no mortality [15] The remaining patients were classified as no TO. Perioperative adherence and complications (classified according to the Clavien-Dindo system) were documented, along with the type of liver resection for each patient. Emphasis was placed on the timing and length of initial and subsequent mobilization (postoperative day [POD] 0–3) for each patient, which was analyzed in relation TO.

### Statistics

Statistical analyses were performed using R (version 4.3; R Foundation for Statistical Computing, Vienna, Austria). Patients were divided into groups according to TO. Analysis between groups was performed using the Wilcoxon rank sum test for continuous variables and Pearson’s Chi-squared test or Fisher’s exact test for categorical variables. For comparison of two continuous variables, Spearman’s rank correlation coefficient was calculated. The significance level (*α*-level) chosen was 0.05.

To identify independent predictors of TO, a multivariable logistic regression model was constructed including key patient (age, body mass index, gender, ASA physical status), preoperative (nutritional status, prior abdominal surgery), surgical (approach), and postoperative (length of stay) factors. Early mobilization was defined as exceeding 3 h on postoperative day 2. Cases with missing data were excluded from this analysis. Model performance was evaluated using Nagelkerke’s *R*^2^. Adjusted odds ratios (ORs) with 95% confidence intervals (CIs) were derived by exponentiating the regression coefficients.

## Results

### Patient demographics

During the study period 1076 patients underwent liver surgery at the Charité – Universitätsmedizin Berlin. The median age across all patients was 63 years (IQR 54–72) years, while patients with TO were significantly younger (62 years vs. 65 years; *p* < 0.001) (Table 1). ASA physical status and preoperative nutritional status did not differ between groups (*p* = 0.058 and *p* = 0.056, respectively). However, more patients with TO had required preoperative stenting (5.4% vs. 21%; *p* < 0.001) and received minimal invasive surgery (74% vs. 40%, *p* < 0.001). Over 80% of patients underwent liver resection for malignant disease, with the most common indications being colorectal liver metastases, hepatocellular carcinoma, and cholangiocarcinoma, while benign indications (e.g., adenoma, focal nodular hyperplasia, cysts) accounted for fewer than 20% of cases. In total, 254 patients (23.6%) were admitted to the ICU postoperatively, with a median stay of 2.0 nights (mean 7.89). Bile leakage occurred in 10.4% of all patients.

**Table 1:** Patient demographics

	Overall *N* = 1076	No textbook outcome *N* = 314^a^	Textbook outcome *N* = 762^a^	*p*-value^b^
Age [years]	63 (54, 72)	65 (57, 73)	62 (54, 71)	< 0.001
BMI [kg/m^2^]	25.4 (22.5, 28.8)	25.7 (22.9, 29.4)	25.2 (22.5, 28.7)	0.10
Sex [f]	489 (45%)	133 (42%)	356 (47%)	0.2
ASA status				0.058
ASA 1	54 (5.0%)	8 (2.5%)	46 (6.0%)	
ASA 2	420 (39%)	119 (38%)	301 (40%)	
ASA 3	589 (55%)	182 (58%)	407 (53%)	
ASA 4	13 (1.2%)	5 (1.6%)	8 (1.0%)	
Preoperative nutritional status				0.056
Normal status	893 (83%)	249 (79%)	644 (85%)	
Risk of malnutrition	124 (12%)	46 (15%)	78 (10%)	
Yes, malnourished	39 (3.6%)	11 (3.5%)	28 (3.7%)	
Previous abdominal surgery	470 (44%)	149 (47%)	321 (42%)	0.11
Preoperative stenting	108 (10%)	67 (21%)	41 (5.4%)	< 0.001
Type of surgery				
Exploratory surgery	50 (4.6%)	7 (2.2%)	43 (5.6%)	
Extended left hepatectomy	81 (7.5%)	41 (13%)	40 (5.2%)	
Extended right hepatectomy	121 (11%)	69 (22%)	52 (6.8%)	
Left hepatectomy	121 (11%)	23 (7.3%)	98 (13%)	
Other	32 (3%)	10 (3.3%)	22 (2.9%)	
Other segmentectomies	424 (39%)	101 (32%)	323 (42%)	
Right hepatectomy	148 (14%)	55 (18%)	93 (12%)	
Wedge or minor resections	99 (9.2%)	8 (2.5%)	91 (12%)	
Surgical approach				< 0.001
Minimally invasive surgery	692 (64%)	126 (40%)	566 (74%)	
Open	384 (36%)	188 (60%)	196 (26%)	
Conversion	46 (6.5%)	18 (8.5%)	28 (5.6%)	0.5
Time to passage of stool [nights]	3 (2, 4)	3 (3, 4)	3 (2, 3)	< 0.001
Time to tolerating solid food [nights]	2 (1, 4)	3 (2, 5)	2 (1, 3)	< 0.001
Length of stay [nights]	6 (5, 10)	15 (9, 25)	6 (4, 7)	< 0.001
Clavien Dindo				< 0.001
Grade I	31 (2.9%)	31 (9.9%)	0 (0%)	
Grade II	71 (6.6%)	71 (23%)	0 (0%)	
Grade IIIa	116 (11%)	116 (37%)	0 (0%)	
Grade IIIb	46 (4.3%)	46 (15%)	0 (0%)	
Grade IVa	21 (2%)	21 (6.7%)	0 (0%)	
Grade IVb	4 (0.4%)	4 (1.3%)	0 (0%)	
Grade V	19 (1.8%)	19 (6.1%)	0 (0%)	
Bile leakage				< 0.001
Grade A	13 (1.7%)	13 (5.0%)	0 (0%)	
Grade B	59 (7.8%)	59 (23%)	0 (0%)	
Grade C	7 (0.9%)	7 (2.7%)	0 (0%)	
Mobilization [Y/N]				
Day of surgery	336 (31%)	55 (18%)	281 (37%)	< 0.001
Postoperative day 1	845 (79%)	210 (67%)	635 (83%)	< 0.001
Postoperative day 2	883 (82%)	227 (72%)	656 (86%)	< 0.001
Postoperative day 3	859 (80%)	236 (75%)	623 (82%)	< 0.001

^a^*n* (%)

^b^Wilcoxon rank sum test; Pearson’s Chi-squared test; Fisher’s exact test

Comparing postoperative recovery between groups, notable differences were observed in solid food tolerance and time to stool passage: the no TO group had a median time of 3 days to tolerate solid food (range of 2–5 days), whereas the TO group tolerated solid food earlier, with a median of 2 days (range of 1–3 days), with *p* < 0.001. TO patients also showed quicker times of passage of stool (*p* < 0.001). The median hospital stay was notably longer in no TO patients (median of 15 Nights; range of 9–25 nights) in comparison to TO patients (median of 6 Nights; range of 4–7 nights) with *p* < 0.001. Significant differences were also observed in the types of surgery performed, such as *extended* left and right hepatectomy being more common in the no TO group.

### Extent of resection influences length postoperative mobilization

**Fig. 1:**
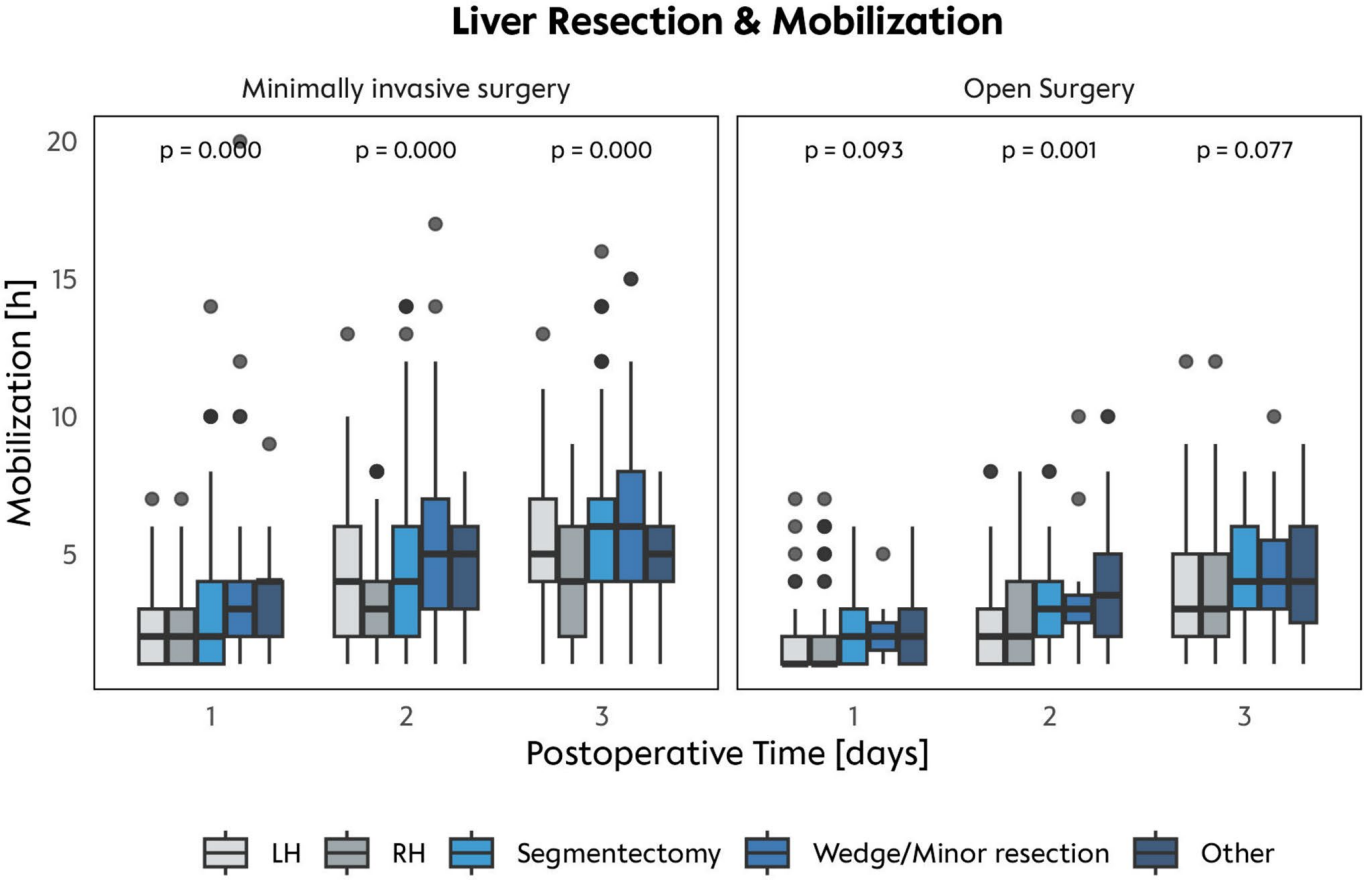
Procedures and mobilization. Mobilization hours per day shown for all liver resection procedures, stratified by MILS and open surgery approaches over POD 1 and 3

**Fig. 2:**
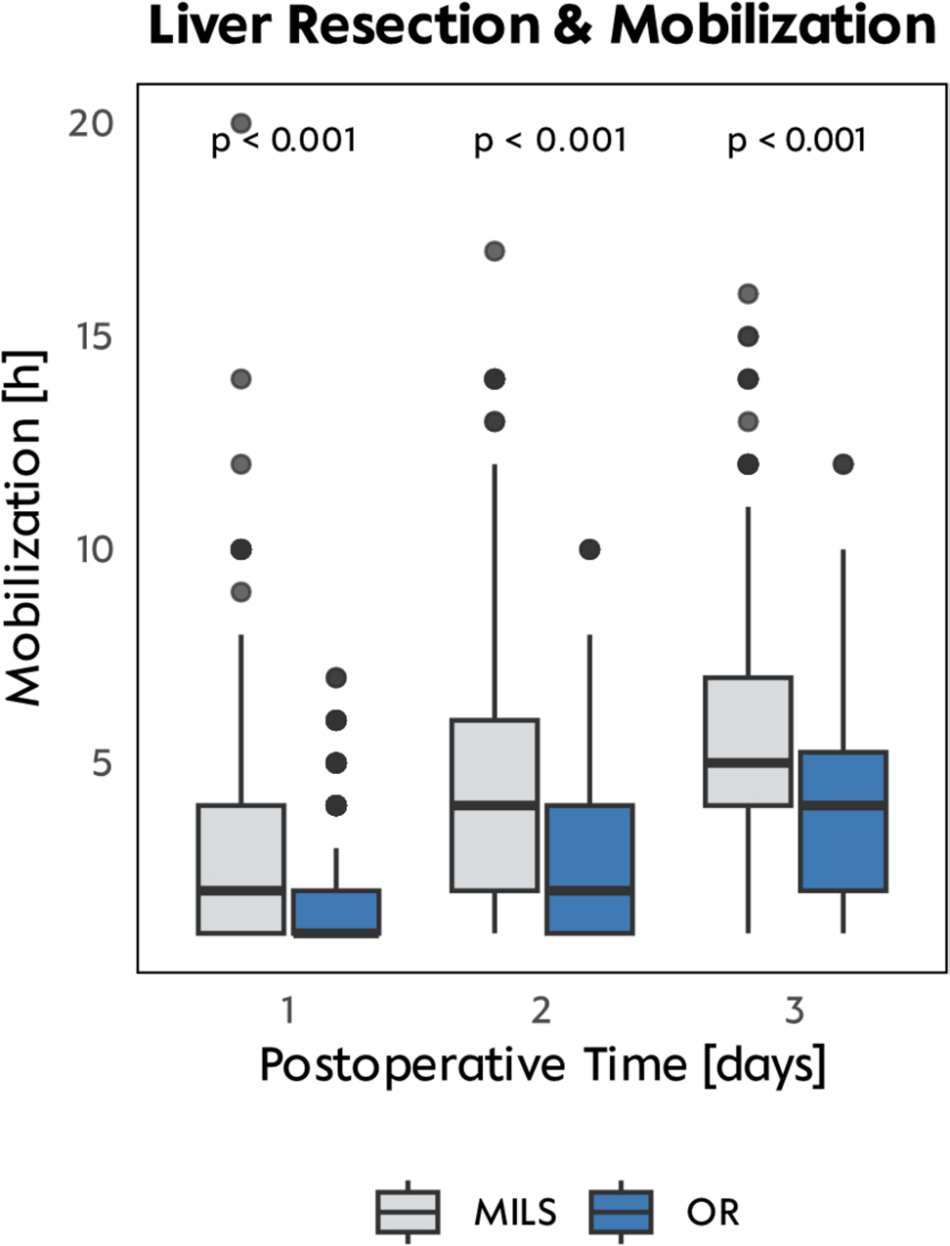
Mobilization on POD 1–3. Overall comparison of minimally invasive liver surgery (MILS) vs open resection over POD 1 and 3

Analyzing postoperative mobilization regarding the extent of surgery, we observed: (1) an increase in mobilization over time (days), (2) higher median mobilization duration in patients after MILS in comparison to OR, and (3) lower median mobilization duration in patients receiving major liver resections. In detail, during the first 2 postoperative days, patients who underwent MILS showed a significant increase in mobilization time, nearly doubling from POD 1 to POD 3 (POD 1: median 2 h, POD 2: median 4 h, POD 3: median 5 h; *p* < 0.001), indicating a steady postoperative improvement (Fig. 1). A TO MILS right hemihepatectomy was associated with significantly shorter mobilization times on POD 1 to POD 3—2 h (IQR 1–3 h), 3 h (IQR 2–4 h), and 4 h (IQR 3–6 h), respectively—compared to a TO MILS segmentectomy, which showed mobilization times of 2 h (IQR 2–4 h), 4 h (IQR 3–6 h), and 6 h (IQR 4–7 h). Overall, MILS patients mobilized more and faster than OR patients: On POD 1, a median of 2 h (IQR 1–4 h) vs. 1 h (IQR 0–2 h); on POD 2, a median of 4 h (IQR 2–7 h) vs. 2 h (IQR 1–4 h); and on POD 3, a median of 5 h (IQR 3–8 h) vs. 4 h (IQR 2–6 h) (all *p* < 0.001) (Fig. 2).

### Defining optimal mobilization durations

**Fig. 3:**
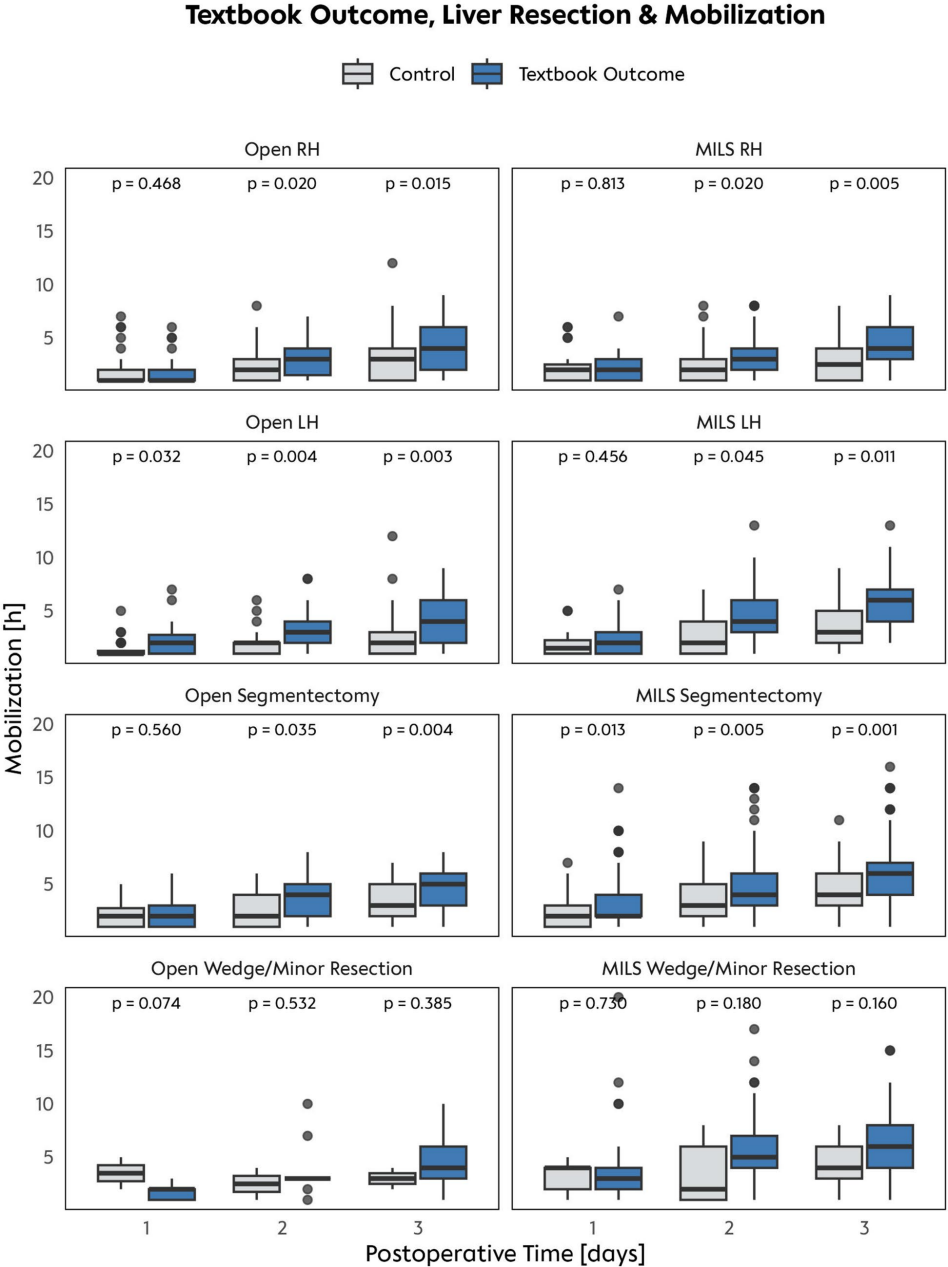
Textbook outcome, liver resection and mobilization. Postoperative mobilization (in hours) is compared over the first 3 postoperative days for all listed types of liver resections. Each boxplot separates patients into TO (blue) and control (gray) groups (Color figure online)

To define optimal cut-off values, patients were categorized into TO and no-TO groups. Indeed, mobilization times varied significantly depending on the type of liver resection performed (Fig. 3). For example, in TO patients who underwent open right hepatectomy, mobilization on POD 2 was 1 h longer compared to no TO patients (median of 3 h [IQR 2–6 h] vs. 2 h [IQR 1–4 h]; *p* = 0.02). Similarly, for minimally invasive segmentectomy, TO patients had two additional hours of mobilization on POD 3 (median of 6 h [IQR 4–10 h] vs. 4 h [IQR 2–6 h]; *p* = 0.001). While wedge or minor resections appeared to be an exception to this trend, the data clearly indicate a strong relationship between increased mobilization length and achieving TO. To determine if these findings could support recommendations for optimal postoperative mobilization lengths, we compared mobilization length by TO in detail (Table 2). Except for minimal invasive minor resections, no differences could be identified on POD 1. However, globally—for all types of liver resections, a median of 2 h for POD 1 (IQR 1–4 h), 4 h for POD 2 (IQR 2–6 h) and 5 h for POD 3 (IQR 4–7 h) were significantly associated with TO (all *p* < 0.001). When analyzing specific subgroups, the largest differences in mobilization intensity were observed for open left hepatectomies on POD 1 and POD 2. Patients with TO mobilized for a median of 2 h (IQR 1–3 h) on POD 1 compared to 1 h (IQR 1–1.5 h) without TO (*p* = 0.032). On POD 2, patients with TO mobilized for a median of 4 h (IQR 2–6 h) compared to 2 h (IQR 1–3 h) without TO (*p* = 0.004). In contrast, the smallest differences were noted in patients undergoing minimal invasive wedge or minor resections. On POD 1, no significant differences in mobilization intensity were detected (median 3 h [IQR 2–4 h] for TO vs. 4 h [IQR 2–4 h] for no TO, *p* = 0.730). On POD 3, these differences remained non-significant, with TO patients mobilizing for a median of 5 h (IQR 4–7 h) versus 4 h (IQR 3–6 h) without TO (*p* = 0.160). Taken together, postoperative complications were associated with shorter mobilization times, and vice versa. The optimal mobilization duration differs significantly between the type of liver resection.

**Table 2:** Postoperative mobilization length

	Postoperative day 1 [h]			Postoperative day 2 [h]			Postoperative day 3 [h]		
	No TO^a^	TO^a^	*p*-value^b^	No TO^a^	TO^a^	*p*-value^b^	No TO^a^	TO^a^	*p*-value^b^
Any liver surgery	1 (1, 2)	2 (1, 4)	< 0.001	2 (1, 4)	4 (2, 6)	< 0.001	3 (2, 5)	5 (4, 7)	< 0.001
Any open resection	1 (1, 2)	2 (1, 2)	0.053	2 (1, 3)	3 (2, 4)	< 0.001	3 (1.5, 4)	4 (3, 6)	< 0.001
Any MILS	2 (1, 3)	2 (1, 4)	< 0.001	2 (1, 4)	4 (2, 6)	< 0.001	3.5 (2, 6)	6 (4, 7)	< 0.001
Open right hepatectomy	1 (1, 2)	1 (1, 2)	0.468	2 (1, 3)	3 (1, 4)	0.020	3 (1, 4)	4 (2, 6)	0.015
MILS right hepatectomy	2 (1, 3)	2 (1, 3)	0.813	2 (1, 3)	3 (2, 4)	0.020	2.5 (1, 4)	4 (3, 6)	0.005
Open left hepatectomy	1 (1, 1.5)	2 (1, 3)	0.032	2 (1, 2)	3 (2, 4)	0.004	2 (1, 3)	4 (2, 6)	0.003
MILS left hepatectomy	1.5 (1, 2.5)	2 (1, 3)	0.456	2 (1, 4)	4 (3, 6)	0.045	3 (2, 5)	6 (4, 7)	0.011
Open segmentectomy	2 (1, 3)	2 (1, 3)	0.560	2 (1, 4)	4 (2, 5)	0.035	3 (2, 5)	5 (3, 6)	0.004
MILS segmentectomy	2 (1, 3)	2 (2, 4)	0.013	3 (2, 5)	4 (3, 6)	0.005	4 (3, 6)	6 (4, 7)	0.001
Open wedge/minor resection	3.5 (2, 5)	2 (1, 2)	0.074	2.5 (1, 4)	3 (3, 3)	0.532	3 (2, 4)	4 (3, 6)	0.385
MILS wedge/minor resection	4 (2, 4)	3 (2, 4)	0.730	2.0 (1, 6)	5 (4, 7)	0.180	4 (3, 6)	6 (4, 8)	0.160

*MILS* minimal invasive liver surgery (laparoscopic or robotic-assisted surgery), *TO* textbook outcome

^a^*n* (%); Median (*Q*1, *Q*3)

^b^Wilcoxon rank sum test

After adjustment, early mobilization on POD 2 was independently associated with higher odds of achieving TO (OR 1.13, 95% CI 1.08–1.19; *p* < 0.001). Undergoing open surgery decreased the odds of TO compared with minimally invasive techniques (OR 0.84, 95% CI 0.80–0.89; *p* < 0.001). Each additional night in hospital was associated with a small reduction in odds (OR 0.99 per Night, 95% CI 0.99–0.99; *p* < 0.001), and each additional year of age reduced the odds by approximately 0.3% (OR 0.997 per year, 95% CI 0.995–0.999; *p* = 0.001). None of the other covariates—gender, BMI, ASA class, nutritional status category, or prior abdominal surgery—showed significant associations with TO (all *p* > 0.05). These findings demonstrate that, beyond patient and procedural factors, early and intensive mobilization on POD 2 is a robust independent predictor of optimal postoperative recovery. Model performance was strong: McFadden’s pseudo‐*R*^2^ was 0.29, Cox & Snell *R*^2^ was 0.28, and Nagelkerke’s *R*^2^ was 0.41, indicating that the full model explained a substantial proportion of variation in TO.

### Impact of surgery timing and duration on complications and mobilization

**Fig. 4:**
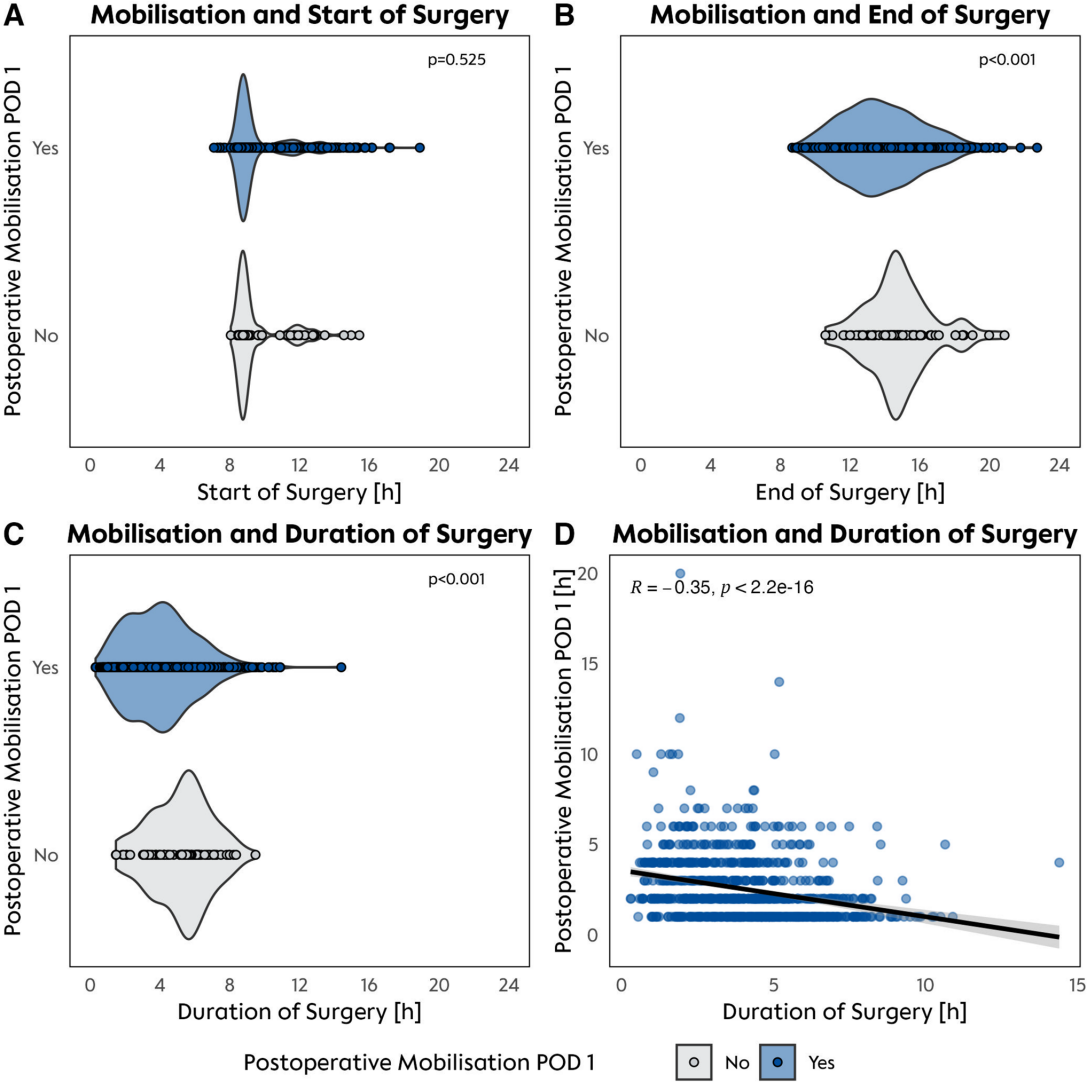
Association between surgery times and mobilization on POD 1. **A** Starting time of surgery. **B** Ending time of surgery. **C** Duration of surgery put in context to occurrence of postoperative mobilization on POD 1. **D** Correlation of mobilization and surgery time on POD 1. Times are measured in hours, levels of significance shown by *p*-value

**Fig. 5:**
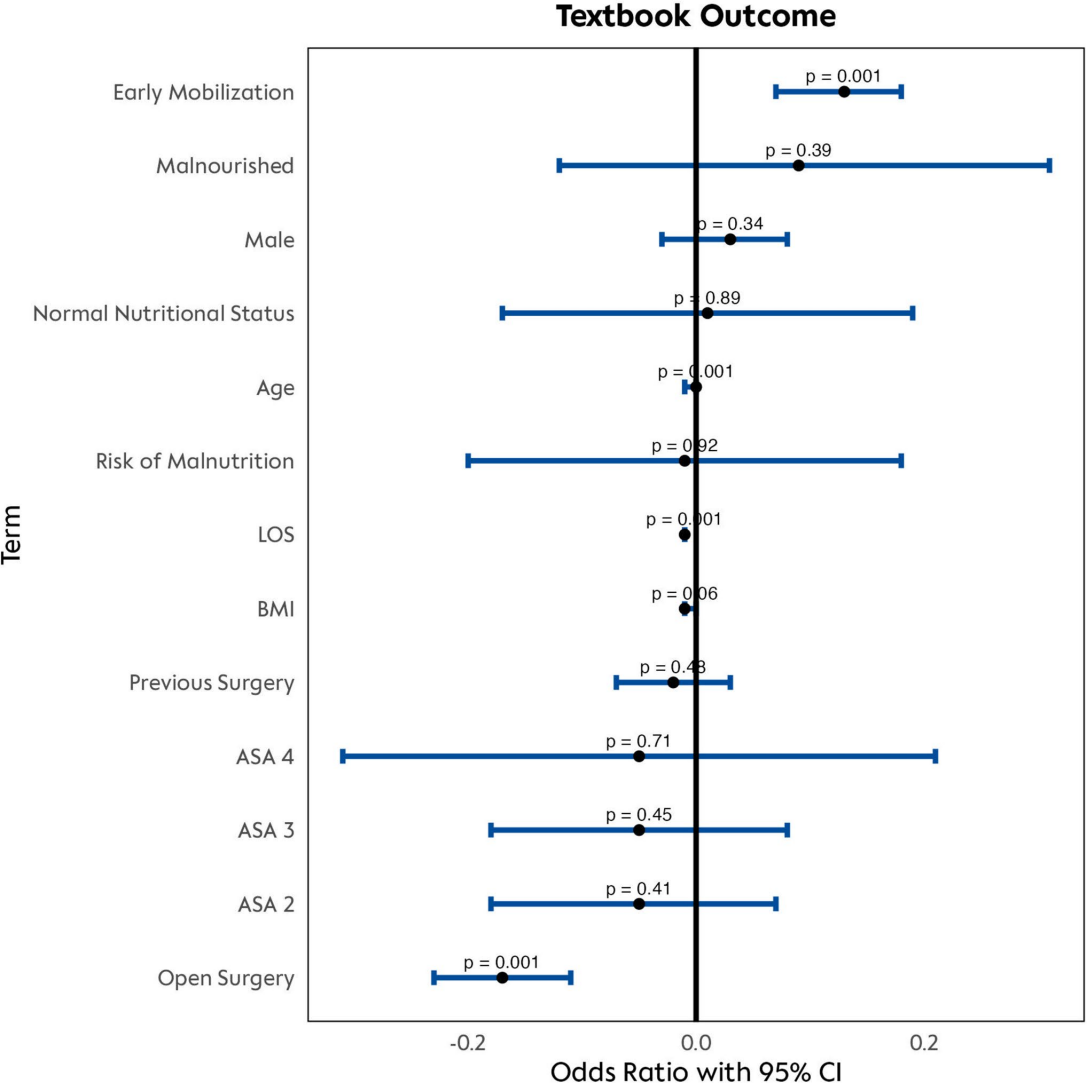
Factors associated with achieving a TO. Multivariable logistic regression analysis of factors associated with TO was conducted, with each line representing a variable and its effect on the likelihood of achieving TO. Black dots indicate odds ratios; blue lines show 95% confidence intervals (Color figure online)

Earlier surgery start times were associated with increased likelihood of mobilization on POD 0. Patients whose surgeries began earlier in the day, with a median start time of 8:50 AM (IQR 8:40–9:25 AM), were significantly more likely to mobilize on POD 0 compared to those with later surgery times (*p* = 0.012). However, this association was no longer observed on POD 1. Patients with earlier surgery start times on POD 1, with a median of 9:10 AM (IQR 8:40–9:55 AM), were not significantly more likely to mobilize compared to those with later start times (*p* = 0.525) (Fig. 4). In contrast, shorter surgery durations, with a median of 3 h and 5 min (IQR 2 h and 0 min to 4 h and 30 min), were closely linked to early mobilization (*p* < 0.001). There was also a moderate negative correlation between surgery duration and total mobilization time on POD 1 (*R* = − 0.35, *p* < 2.2e–16) (Fig. 5).

## Discussion

This is the first large cohort study to evaluate mobilization following liver resection within the framework of a structured ERAS program. Not only was it demonstrated that differences exist between patients with and without complications (TO vs. no TO), but significant differences were also observed based on the complexity of the procedures. For example, a patient undergoing a laparoscopic wedge resection achieves significantly better mobilization compared to patients undergoing an extended open right hemihepatectomy. This aspect is currently not addressed in the ERAS recommendations by the ERAS Society and should be considered for future guideline updates [12]. Possible cut-offs for mobilization on POD 1 to POD 3 can be determined based on the data presented in Table 2.

Early mobilization is a cornerstone of the ERAS protocol and is supposed to reduce postoperative complications [16]. The association between higher mobilization times and achieving TO is striking in our study. On POD 2 and 3, TO patients were mobilized significantly more than no TO patients. The benefits of early mobilization are well-documented in various surgical fields, including liver surgery. Mobilization prevents respiratory complications by encouraging deeper breathing and improving lung function [17, 18], thus reducing the risk of atelectasis and pneumonia. Furthermore, it promotes circulation, decreasing the risk of venous thromboembolism [19, 20] and it has a positive impact on gut motility, lowering the rates of gastrointestinal complications [21–23]. Several factors contribute to the ability to mobilize early after surgery, including multimodal pain management and preoperative patient education, all of which are integral to our ERAS protocols [12]. Multimodal analgesia, including regional anesthesia and non-opioid pain control, plays a vital role in enabling patients to mobilize early by reducing postoperative pain without the sedative effects of opioids [24, 25]. However, it must be pointed out that it remains unclear what influence the duration of mobilization has on the patient’s outcome. We cannot definitively determine whether patients were not mobilized due to complications and their associated complication management (endoscopy, imaging, redo surgery), or whether poor compliance and reduced mobilization actually contributed to a higher complication rate. This question can only be addressed and answered through a randomized controlled trial.

In colorectal surgery, where ERAS protocols have been widely implemented and studied, early mobilization has similarly shown strong associations with improved outcomes, including reduced length of stay and fewer complications. Studies [26, 27] highlight the importance of mobilization within the first 24 h after surgery as a key predictor for the course of recovery. Compared to these colorectal cohorts, our liver surgery data show similar trends, despite the higher complexity and variability of hepatic resections. This supports the notion that early mobilization is a universal ERAS principle with benefits across surgical disciplines. However, specific adaptations, such as accounting for the invasiveness of liver surgery, need to be considered.

Minimally invasive surgical techniques, such as laparoscopic liver resections, have also been shown to facilitate earlier mobilization. These techniques are associated with less postoperative pain, lower surgical stress, and faster recovery times compared to traditional open surgery [28]. Our analysis supports these claims, revealing that MILS were more frequently performed in TO patients. Conversely, extended resections and open surgeries were more common in the group, highlighting the increased risk of complications in these cases. MILS patients achieved significantly higher median mobilization times, particularly on POD 2 and 3. For example, patients undergoing MILS segmentectomy mobilized for a median of 4 h on POD 2 and 6 h on POD 3, compared to 3 and 4 h, respectively, in open segmentectomy. This early mobilization was associated with a shorter hospital stay by a median of 9 days; however, this relationship may be influenced by other perioperative factors.

The overall complexity of the surgical procedure also played a role in outcomes. Extended left and right hepatectomies, which are more commonly performed via open surgery, were associated with lower TO rates and longer recovery times, while wedge or minor resections, particularly when performed minimally invasively, showed the highest rates of TO. Further analysis revealed the influence of surgery timing and duration on early mobilization and postoperative outcomes. Patients whose surgeries began earlier in the day (median start time approximately 8:38 am) were more likely to achieve mobilization compared to those with later surgery start times (*p* = 0.012). This may reflect both the greater energy and readiness of the medical team during morning surgeries, as well as the increased window of opportunity for postoperative interventions, such as mobilization, in the later hours of the day. Surgery duration also emerged as a significant factor, with shorter surgeries being linked to a higher likelihood of early mobilization on POD 0 (*p* < 0.001). Patients who underwent shorter surgeries (median duration of 2.78 h) were more likely to mobilize on POD 0 compared to those with longer surgeries (median 5.33 h), where complication rates were notably higher (*p* < 0.001). Prolonged surgeries may contribute to greater surgical stress, prolonged anesthesia, and a higher incidence of complications, which can delay recovery and reduce the ability to mobilize early [29].

Preoperative education and empowerment of the patient and their relatives is another critical factor in promoting early mobilization. Patients who are well-informed about the importance of early movement and the expectations for postoperative recovery are more likely to engage in mobilization early in the recovery process. Simultaneously, all involved parties (surgeons, nurses, physiotherapists) should be sensibilized regarding all ERAS modules. This awareness, combined with the supportive environment of the ERAS protocol, encourages patients to participate actively in their recovery [23].

Even when the clinical team recognizes the benefits of early mobilization and believes they outweigh the perceived risks, a recurring issue is the shortage of staff to help patients engage in out-of-bed activities [30]. Studies found that patients were significantly more likely to mobilize when a physical therapist was present [31]. Ideally, especially in high-acuity postoperative settings, support from physical therapists should be a part of usual care, starting within 24 h after surgery. Our results indicate that tailored interventions are needed to support early mobilization to achieve TO. For example, enhanced pain management strategies or the use of assistive devices for early movement could help bridge the gap for patients who are unable to mobilize on their own. Moreover, the role of prehabilitation—strengthening patients before surgery to improve postoperative outcomes—should be explored as a strategy to enhance the ability to mobilize early, especially in patients with higher preoperative risk factors [32, 33].

Although our study provides valuable insights into the importance of early mobilization, several limitations should be noted. With a prospective design, but single-center nature of the data, the generalizability of our findings may be restricted. As an observational study, this analysis cannot establish causality between mobilization and outcomes like TO. Patients with TO had more favorable baseline and surgical profiles, and unmeasured confounders, such as comorbidities, fluid management, nutritional intake, or complications, may have influenced both mobilization and outcomes. Additionally, data on ICU stay was not part of the primary analysis and data were not collected systematically, thus limiting the interpretability of the descriptive numbers reported. Future prospective studies are needed to validate these results and refine mobilization protocols, particularly regarding the optimal timing and duration of mobilization. Furthermore, the integration of objective metrics, such as wearable activity trackers, could provide more precise assessments of mobilization length and its impact on recovery.

## Conclusion

Our findings underline a strong association between increased postoperative mobilization and favorable outcomes following both easy and complex liver resections. Notably, patients who experienced complications tended to mobilize less; however, this does not imply a causal relationship, and it remains unclear whether longer mobilization durations can prevent complications.

## Abbreviations

ASA: American Society of Anesthesiologists
BMI: Body mass index (kg/m^2^)
CD: Clavien-Dindo
ERAS: Enhanced Recovery After Surgery
IQR: Interquartile range
MILS: Minimally invasive liver surgery
POD: Postoperative day
TO: Textbook outcomes in liver surgery
